# Primary prevention of overweight in preschool children, the BeeBOFT study (breastfeeding, breakfast daily, outside playing, few sweet drinks, less TV viewing): design of a cluster randomized controlled trial

**DOI:** 10.1186/1471-2458-13-974

**Published:** 2013-10-19

**Authors:** Hein Raat, Mirjam K Struijk, Teun Remmers, Eline Vlasblom, Amy van Grieken, Suzanne ML Broeren, Saskia J te Velde, Maaike Beltman, Magda M Boere-Boonekamp, Monique P L’Hoir

**Affiliations:** 1 Department of Public Health, Erasmus MC - University Medical Center Rotterdam, Dr. Molewaterplein 50, PO Box 2040, 3000 CA Rotterdam, the Netherlands; 2 TNO Child Health, Leiden, the Netherlands; 3 Division of Science, Technology, Health and Policy Studies, University of Twente, Enschede, the Netherlands; 4 EMGO+ Institute for Health and Care Research and the Department of Epidemiology and Biostatistics, VU University Medical Center, Amsterdam, the Netherlands

## Abstract

**Background:**

Two overweight prevention interventions were developed to be offered by preventive Youth Health Care (YHC) in addition to the currently applied overweight prevention protocol to parents of 0-3 year old children. The two interventions aim to support parents of preschool children to realize healthy child nutrition and activity behaviors of their young child. The aim of this study is to assess the effects of the two overweight prevention interventions with regard to child health behaviors and child Body Mass Index.

**Methods/Design:**

A cluster randomized controlled trial was conducted among parents and their preschool children who attend one of 51 participating YHC teams. The teams were randomly allocated to one of the two intervention groups, or to the control group (care as usual).

The ‘BBOFT+’ intervention focuses on effective child rearing by parents from birth onwards by enlarging parental skills concerning healthy behavioural life-style habits. Parents who are allocated to the ‘E-health4Uth Healthy toddler’ intervention group, at the child age of circa 18 and 24 months old, are invited to complete an online E-health module providing tailored health education regarding healthy child nutrition and activity behaviors. The E-health messages are discussed and reinforced during the subsequent regularly scheduled visits by YHC professionals, and were repeated after 4 weeks.

The primary outcome measures at child age 3 years are: overweight inducing/reducing behaviors, (for ‘BBOFT+’ only) healthy sleep, Body Mass Index and prevalence of overweight and obesity. Secondary outcome measures are attitudes and other cognitive characteristics of the parents regarding the overweight-related behaviors of their child, parenting styles and practices, and health-related quality of life of the children.

**Discussion:**

We hypothesize that the use of the additional interventions will result in a healthier lifestyle of preschool children and an improved BMI and less development of overweight and obesity compared to usual care.

**Trial registration:**

Nederlands Trial Register NTR1831.

## Background

In the Netherlands, a system for monitoring children’s health and development, and for providing health promotion and disease prevention at set ages from birth onwards is available: i.e. preventive Youth Health Care (YHC), which is offered nation-wide and free of charge [1]. Participation is voluntary and the attendance rate is about 95%. During the YHC visits, growth and development of the child are assessed [1,2]. YHC is committed to counsel parents regarding parenting competence, and to promote healthy development and growth for all children [2]. YHC can also support public health strategies that are needed to tackle the problem of the increasing prevalence of childhood overweight and obesity [3,4]. In 2010, the prevalence of overweight (including obesity) in the Netherlands was 13,3% and 14,9% for 2–17 year-old boys and girls respectively. For obesity, this was 1,8% for boys and 2,2% for girls [5].

Consensus regarding overweight prevention in the setting of YHC has been reached and consists of two elements, i.e. a YHC-Overweight detection protocol [6] and a YHC-Overweight prevention protocol [7]. The YHC-Overweight prevention protocol includes (a) a strategy for primary prevention of overweight (i.e. promotion of healthy energy balance-related behaviors in the child population), and (b) a strategy for secondary prevention of overweight (i.e. detection of children with overweight and intensive counseling of these children and their parents).

Based on the YHC-Overweight prevention protocol and an observational study that included 390 families with children aged 0–4 years regarding health behaviors and parents’ views regarding health behaviors of their young children [8], we developed two interventions, i.e. the ‘BBOFT+’ and the ‘E-health4Uth Healthy toddler’ interventions. The ‘BBOFT+’ intervention focusses on effective child rearing by parents from birth onwards by enlarging parental skills concerning healthy behavioural life-style habits of the child. The ‘E-health4Uth Healthy toddler’ intervention consists of an E-health module providing tailored health education regarding healthy child nutrition and activity behaviors, which is combined with face to face counseling by YHC nurses.

The two interventions, ‘BBOFT+’ and ‘E-health4Uth Healthy toddler’ both fit into the YHC Overweight-prevention-protocol. Both interventions aim to promote relevant health behaviors regarding a healthy weight development of young children: breast feeding (only in the ‘BBOFT+’ intervention), daily exercise/outdoor playing, breakfast daily, few sweet drinks, and minimal TV time. Additionally, the ‘BBOFT+’ intervention is designed to promote healthy sleep behaviors of the child. The two interventions are described in detail below.

### Objective

In this study, the effectiveness of the two interventions (‘BBOFT+’ and ‘E-health4Uth Healthy toddler’) that involve primary prevention of overweight and obesity in early childhood is evaluated in comparison with usual care in a cluster randomized controlled trial. In addition, the level of adherence of parents and YHC professionals to the distinct elements of the ‘BBOFT+’ intervention and the ‘E-health4Uth Healthy toddler’ intervention will also be taken into account, as well as how parents appreciate these elements. In this article the design of the evaluation study is described.

### Study hypotheses

The hypotheses of this study are that at the age of three years, children in the two intervention groups will have healthier lifestyles and a more favorable development of BMI, i.e. less development of overweight and obesity, compared to children in the control condition (‘usual care’). In addition, we hypothesize that attitudes and other cognitive characteristics of the parents regarding the overweight-related behaviors of their child, and that parenting styles and practices, and health-related quality of life of the children will be more favorable in the two intervention groups compared to the control condition.

## Methods/Design

### Study design, procedure and participants

**Figure 1:**
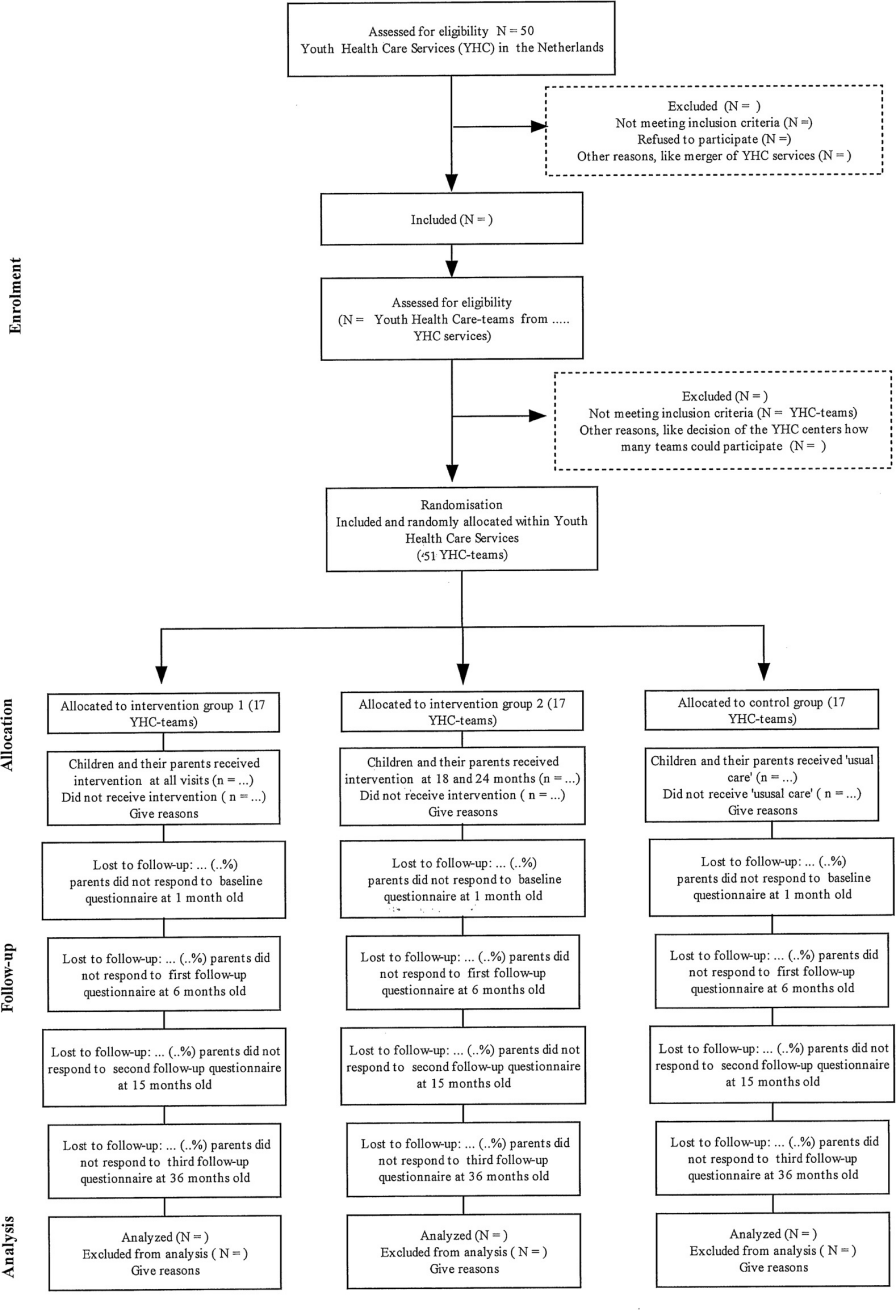
Flowchart of the design of the study.

The study is a cluster randomized controlled trial (c-RCT) with two intervention conditions and a control condition (‘usual care’). The inclusion of participants is shortly after child birth, with repeated measurements; the last measurements occur at child age of circa 36 months (see Figure 1).

Managers of all regional Youth Health Care (YHC) organizations in the Netherlands (n=50) were informed about the study and were invited to participate in the study. Ten YHC organizations volunteered to participate. In total, 51 YHC teams were available to participate in the study (three times seventeen teams). These teams covered both rural and urban regions in the Netherlands. Prior to the start of the study, the researchers arranged meetings to explain the procedure of the study, and to instruct the participating YHC professionals. Within each organization, YHC teams were randomly assigned to either one of the two intervention groups or the control group by a computerized random allocation generator. Data collection started in 2008 and continues until fall 2013.

The Medical Ethical Committee of Erasmus Medical Center has declared that the Medical research Involving Human Subjects Act (also known by the Dutch acronym WMO) does not apply to this study. The Medical Ethical Committee had no objection against the execution of this research proposal (reference number MEC-2008-250).

The study includes parents and their babies/toddlers that belong to one of the participating YHC teams, and who are eligible for the regular YHC visits from birth until age three years. The parents are informed about the study when the YHC nurse visits the parents in the second week after birth. Parents are provided with information about the study, an informed consent form, and a short questionnaire with items on background data (i.e. the baseline questionnaire); the parents were invited to provide written informed consent to participate in the study, and to complete the short questionnaire. The study design and participant flow are shown in Figure 1.

### The ‘BBOFT+’ intervention

The ‘BBOFT+’ intervention intends to promote the key behaviors for the prevention of overweight in children as defined in the YHC-Overweight Prevention Protocol (see Table 1); i.e. breast feeding, and, once applicable, daily exercise/outdoor playing, breakfast daily, few sweet drinks, and minimal TV time. Additionally, the ‘BBOFT+’ intervention is designed to promote healthy sleep behaviors of the child. Parental positive child rearing competences and skills are addressed in this intervention. YHC professionals receive training about child rearing and aspects of the learning theory to be applied in the course of the ‘BBOFT+’ intervention.

**Table 1:** Targets of the ‘BBOFT+’ approach

Enlarging parenting skills is operationalized as:	- Increase children’s self-esteem by spending time with the child, talk and show affection to the child. Regularly interact with a child while it is behaving positive, and talk with children about their activities, cuddle, touch, and hold the child (body contact)
	- Encourage healthy behaviors: reinforce positive behavior by praising the specific behavior
	- Give positive attention: offer non-verbal attention (smiling, touching)
	- Offer attractive activities: play material etc. (age and development phase related)
	- Parent is positive example (modeling)
	- Reinforce spontaneous learning moments
	- Reinforce learning by experience, instruction
Stimulus control	- Basic rules and regulations (age en development phase related)
	- Direct intervention when child is misbehaving
	- Clear instructions in a quiet way
Operant conditioning	- Misbehavior will be related to direct consequence
	- Time out
	- Positive reinforcement
	- Child rearing: warm, positive, and also monitoring and setting borders
	- Grandparent’s behavior (no spoiling)
Optimize nutrition is operationalized as:	- Establishing eating routine (0–9 months regularity and stimulus reduction)
	- > 6 months specific times for meals and snacks (stimulus control)
	- Prevention of introduction of extra foods (unhealthy snack foods, take away, sugar drinks)
	- Provision of healthy alternatives
Optimize physical activity is operationalized as:	- Prevention of sedentary activities (TV etc.)
	- Improve daily physical activity (daily walk, play-pen, tummy time, play outside etc.)
Optimize sleep duration and sleep behavior is operationalized as:	- Optimal sleep duration per day
	- Hours an infant can remain awake
	- Time the child needs to fall asleep
	- Number of times the child wakes up during the night

The YHC nurses and physicians receive a small, calendar-like booklet, with on the front side pictures of infants and toddlers who show the desired healthy behaviors and on the backside items about the behavioral items to put forward by YHC professional. This booklet is placed on the desk, with the pictures to the parents and the text to the health care worker; the purpose is to support the counseling, especially for those who are low- or illiterate. The items are adapted to the developmental phase of the child and concern: (1) So-called ‘authoritative’ child rearing and parenting skills, (2) Healthy food and feeding behaviors, (3) Daily exercise and outside playing, (4) Minimal television and computer use, (5) Adequate sleep duration and healthy sleep behaviors (see Table 2). All items are emphatically and positively formulated.

**Table 2:** Schematic overview of the ‘BBOFT+’ intervention

Feeding	• Breastfeeding	± 2 weeks
	• Variation in maternal food	± 1,2 months
	• No extra bottle when breast feeding	± 2 weeks, ± 2 months
	• Left overs allowed in bottle	± 2 weeks, ± 2 months
	• No extra supplements in bottle	± 1 month
	• Level spoon for bottle feeding	± 1 month
	• Do not reward every cry or fuss with feeding	± 2 months
	• Eat in a social setting (not in front of TV)	± 1 month
	• Accustom to different structures	± 3 months
	• Accustom to different tastes	± 4, 71/2 months
	• Difference home-made food and food from jars	± 6 months
	• Eating at the same time at the table	± 4,6, 71/2 months
	• No TV watching while eating	± 9,11,18,24,36 months
	• Positive atmosphere at the table	± 9,11,18,36 months
	• Child may eat less or more in this phase	± 14,24 months
		± 14,24 months
Space to move and play with pleasure	• Tummy time	± 2 weeks, 1,2,3,4,6 months
	• Car seat is for transport	± 4 months
	• Not too long in rocking chair	± 4 months
	• Get baby out of playpen before it starts to cry	± 4 months
	• The playpen is a safe a nice place to play	± 6, 71/2,11 months
	• The playpen is a stimulant for motoric development	± 6, 71/2,11 months
	• Let the toddler walk itself when/where possible	± 14,18,24,36 months
Daily outside		± 2 weeks, 1,2,3,6,9,11,14,18 months
Sleep	• Duration (sleeping/awake)	± 2 weeks, 1,2,3,4,6,71/2,9,11, 18,24,36 months
	• Put the baby to sleep awake	± 2,3, 71/2 months
	• Sleep in afternoon gradually reducing	± 71/2 months
	• Late feeding in the evening not necessary anymore	± 71/2,9,11,18 months
	• Children like rituals when going to bed	± 9,11,24,36 months
	• A bottle “to fall asleep” is not necessary	± 9,11,14 months
	• Bedtime	± 11,18,24,36 months
Regularity, uniformity in daily care and reduction of stimuli	• Fixed order: sleeping, feeding, playing, getting tired, bring to bed awake	± 1,2,11,14,18 months
	• Crying increases till 6-8 weeks, and decreases after 8 week	± 1 months
	• Play in playpen, transport in car seat	± 2 months
Parenting	• Role/influence grandparents	± 2,71/2,18,36 months
	• Children need warmth, love and safety	± 2,3,71/2 months
	• Sensitive and warm parenting and at the same time restriction	± 71/2,9,14,18,24,36 months
	• Sweets/food not to be used as reinforcer of behavior	± 71/2,9,14,18 months
	• Parenting style	± 9,24 months
	• Children like predictability	± 14 months
	• Screen/food not to be used as reinforcer of behavior	± 24,36 months
Screen	• A baby and TV watching don’t match	± 3,4,6,71/2,9 months
	• No television in bedroom of the child	± 11,14,18,24,36 months
	• Watch TV together	± 14 months
	• Watch TV/play computer together, not longer than 1 hour, daily	± 24,36 months
Drinking	• When thirsty, offer water	± 4,6,9,11,18,24 months
Snack	• Water and diluted fruit juice or tea (no sugar), and bread crust or cracker	± 9,11 months

Parents who are allocated to a ‘BBOFT+’ team receive advice and care during the regularly scheduled visits, i.e. 8 to 11 contacts in the first year, and 5 contacts in total in the second and third year of life. BBOFT+ YHC nurses and physicians apply the intervention during each contact with parents. Promotion of continued breastfeeding has a specific context and is mainly limited to the first year of life. The other behaviors and child-rearing issues receive attention from birth onwards.

The rationale of the intervention is that parents will enlarge their parenting skills and will increase children’s self-esteem, and will encourage healthy child behaviors by setting a good example and using praise and reward, and by managing problem behaviors of their child by setting ground rules, clear instructions and the use of consistent measures [9,10].

In the ‘BBOFT+’ intervention, YHC nurses have to be able to disseminate knowledge; i.e., they need to have the skills to coach parents in achieving competent parenting. In this intervention, behavioral goals are set out: these goals are partially derived from an earlier study [8]. The intervention targets parenting skills in the context of everyday, naturally occurring examples. Specific anticipating parenting education is given during the standard YHC visits. Parents are taught the principles of stimulus control, modeling and operant conditioning. The ‘BBOFT+’ intervention includes targeted education that anticipates on common problems, and explains the basics of stimulus control and classical conditioning in order to enable parents to create the conditions that stimulate the desired behavior in the child (Table 1).

The start of the intervention focuses on the transition to the phase of a young parent; during this phase, parents are expected to be receptive to suggested behavior changes and new knowledge. The BBOFT+ intervention acknowledges the possibility of enhancing self-control of the child by a so-called ‘authoritative’ parenting style. Modeling, stimulus control, and operant conditioning such as intermittent confirmation are used during these contacts [11]. In BBOFT+, social learning principles are applied consisting of both classic and operant conditioning. For instance that specific behavior is caused by antecedent factors, that one should try to intervene before the unwanted behavior occurs, and that one should create conditions suitable for positive behavior. Parents learn that it is important to set clear boundaries, and to have unambiguous rules. They receive suggestions to help structuring time and space, to introduce ground rules, and to state these rules clearly and consistently. Couples should collaborate as a team in defining the rules and applying them consistently. By controlling the stimuli in this manner, disruptive behavior can be avoided according to this approach [12]. BBOFT+ supports the creation of ‘authoritative’ environments characterized by expression of warmth and emotional support, together with using clear, bidirectional communication [13]. The literature shows that an authoritative child rearing style is related to a lower risk of overweight and obesity [14].

In the BBOFT+ intervention teams, the YHC professionals are offered training to provide them with knowledge, skills and explicit guidelines on child rearing; they are educated about aspects of the learning theory. The introductory training of the ‘BBOFT+’ intervention teams consists of 1 session of 4 hours. After a year, this training is repeated to motivate the ‘BBOFT+’ intervention teams and to ensure proper compliance to the protocol of the intervention (see Table 2). The second training session focuses on the developmental stage of the children at that moment. The trainer of the intervention team is a psychotherapist, educated in behavioral therapy (MH).

### The ‘E-health4Uth Healthy Toddler’ intervention

The ‘E-health4Uth Healthy Toddler’ intervention provides tailored health education for the parents regarding the key behaviors related to childhood overweight prevention, at child age 18 and 24 months. These key behaviors are: (1) Promotion of daily exercise and outdoor playing, (2) Promotion of having breakfast daily, (3) Discouragement of drinking sweet drinks, and (4) Discouragement of TV viewing and computer use, including discouragement of playing computer games [7]. One month prior to the regular YHC visit at child age 18 months, parents receive an invitation to visit the ‘E-health4Uth Healthy Toddler’ website to obtain tailored parenting and health information prior to the YHC-visit, which can be used by the parents to prepare for the face-to-face visit at the YHC office (Table 3). During the counseling session, parents and the YHC professional discuss any issues that arose from the tailored feedback; the YHC professional, with permission of the parents, also has access to the results of the ‘E-health4Uth’ online assessment and tailored information. Approximately one month after the face-to-face counseling, the parents receive a reminder by email including the tailored advice. At child age 24 months, i.e. approximately one month prior to the next regular visit to the YHC center, parents allocated to this intervention group again receive an invitation to visit the ‘E-health4Uth Healthy toddler’ website to obtain tailored information prior to the YHC-visit (Table 3). This feedback again is discussed during the YHC-visit. Circa one month after the second face-to-face counseling, parents receive a reminder by email including the tailored advice.

**Table 3:** Schematic overview of the ‘E-health4Uth Healthy Toddler’ intervention

**Steps according persuasion-**	**Element of the ‘Healthy**		**Age of the**
**communication model [**[20]**]**	**Toddler’ intervention**	**Content/activities**	**child**
Attention for the health message	1^st^ E-health module	- Assessment of four key behaviors	± 18 months
		- Assessment of parenting behavior	
		- Feedback on four key behaviors, in comparison with recommendations	
		- Tailored advice, taking into account the four behaviors and parenting practices	
Changing determinants and behavior:	Counseling with motivational interviewing technique	- Making parents aware of their child’s behavior	± 18 months
		- Motivate parents to prevent overweight in their child	
- Attitude			
- Self-efficacy		- Stress the parents responsibility and importance of strict parenting	
- Parenting behavior		- Motivate parents to change their parenting	
		- Discuss advantages of strict parenting	
		- Discuss potential barriers (e.g. time constraints)	
		- Improve self-efficacy	
		- Provide parenting skills:	
		- Setting rules, giving good examples, use praise and award, do not use food as a means of control	
Maintain behavior change	Reminder	- Reminder of tailored advice from E-Health module	± 18 months
		- Positive feedback	
	2^nd^ E-health module	- Assessment of four key behaviors	± 24 months
		- Assessment of parenting behavior	
		- If behavior change was achieved, parents receive positive feedback. If not, parents receive tailored advice	
	2^nd^ Counseling with motivational interviewing technique	- Assessment of behavior	± 24 months
		- If behavior is improved, provide positive feedback. If not, repeat activities described in 1^st^ counseling	
	2^nd^ Reminder	- Reminder of tailored advice from E-Health module	± 24 months

The intervention is based on the social-ecological model [15], which recognizes the importance of environmental and personal factors on health behavior. In very young children, parents can be considered as the main mediators of the toddler’s environment. According to this model, parents influence the social and physical environment through their general parenting style [16] and parental practices. Fostering an ‘authoritative’ parenting style has shown to decrease food stimuli at home [17]. Therefore, parenting is addressed in order to change the toddler’s behavior. Important determinants of parental behavior can be found in classical behavior change theories, such as the Theory of Planned Behavior [18] and Social Cognitive Models [19]. These theories include attitude, social support, self-efficacy, perceived barriers, knowledge, and awareness. The ‘E-health4Uth Healthy toddler’ intervention aims to create parental awareness of potential future overweight related problems, and motivate parents to prevent the development of overweight in their children from early age on by improving the four key behaviors defined in the YHC-Overweight Prevention Protocol. Positive attitudes of the parents towards applying strict behavior and positive attitudes towards the specific, recommended, parental practices are promoted. Furthermore, parents are supported to overcome barriers for not applying these practices, and the intervention promotes specific skills in order to be able to change their parenting according to the recommendations of the YHC-Overweight Prevention Protocol [7].

According suggestions proposed by McGuire for successful communication, several steps are required in order to achieve behavior change [20]. First, the prevention of potential/future overweight related problems have to be brought to attention and made understood. Second, determinants of the behavior have to be changed (e.g. create a positive attitude towards the recommended behavior, and increase self-efficacy to perform the behavior). Finally, behavior change has to be maintained using feedback and reinforcement. The ‘Healthy toddler’ intervention package follows this scheme by application of the different elements included in this intervention package (see Table 3).

At the child age 18 months, the E-health module is introduced to the parents along with the regular invitation for the scheduled YHC visit. This brings the topic to the attention of parents, prior to visiting the YHC center. Results from a pilot study with the ‘E-health4Uth Healthy Toddler’ showed that the majority of the parents indeed understand the message and view this message as useful and applicable. During the subsequent face-to-face counseling at the YHC center, the YHC professional increases knowledge, awareness, and addresses attitudes and self-efficacy with regard to relevant parenting practices. The YHC professionals apply motivational interviewing, which has proven to be effective in changing health behaviors in various settings and which has been adapted for this specific YHC application [21]. Finally, approximately one month after the face-to-face counseling at the YHC center, parents receive an electronic reminder regarding their tailored advice, also including this tailored advice. The intervention is repeated at the child age 24 months.

The ‘E-health4Uth Healthy Toddler’ intervention is delivered to the parents through an Internet-based E-health module. This module has the potential of reaching almost all parents, as 94% of the Dutch households have Internet access [22]. This E-health module assesses the four priority behaviors defined by the YHC-Overweight Prevention Protocol, the general attitudes of the parents toward overweight prevention from early age on, and whether parents apply the recommended parental practices. Participants receive feedback tailored to their individual situation given the provided answers posed by the E-health module online. As part of the tailored feedback, current behavior is compared to the recommendations for each of the four key behaviors. This promotes awareness of the parents regarding their behaviors.

Additionally, parents receive tailored advice on how to change their child’s behavior (e.g., setting rules) [7]. The information entered by the parents regarding the four key behaviors and a summary of their attitudes generated by the E-health module is also available to the YHC professional prior to the visit, enabling the professional to use this information in the counseling session.

YHC professionals in the ‘E-health4Uth Healthy Toddler’ intervention groups are trained to use the intervention and to apply motivational interviewing [23] in the context of the YHC overweight prevention protocol during a half-day session prior to the start of the intervention. This training is given by a certified trainer in motivational interviewing techniques, as applied in preventive health care. Training is performed in groups of 10–25 YHC professionals. The following elements are included in the training: (1) Stimulation for making parents aware of the risk for overweight and its potential adverse effect for their child, (2) Stimulation for favorable change of parental attitudes and motivations regarding overweight prevention and authoritative parenting, (3) Stimulation for the parent’s perception of being responsible for the health of their child, (4) Stimulation for goal setting and providing feedback regarding realistic goals that may increase authoritative parenting and improve energy balance-related behaviors in their child, (5) Providing information on beneficial parental practices and stimulating to teach parents these skills, and (6) Providing information on useful sources of information for parents.

### The control condition: care as usual

The control condition consists of parents of preschool children that participate in the study and receive preventive Youth Health Care from one of the YHC teams that apply ‘usual care’ (i.e., control condition). Usual care includes the regular scheme of YHC visits. The YHC professionals in the control condition are aware of the principles of the Overweight Prevention Protocol; i.e. the importance of healthy lifestyles for the prevention of overweight and obesity (promotion of breast feeding, daily exercise and outdoor playing time, having breakfast daily, lessening sweet drinks, and limiting TV time during their contacts with parents and children) [2,7]. However, in the control condition the YHC professionals did not receive any specific training regarding overweight prevention (as was provided in the ‘BBOFT+’ condition and in the ‘E-health4Uth Healthy Toddler’ condition), no specific supporting materials were available such as a calendar-like booklet on the desk of the professional with pictures to support counseling (as was available in the ‘BBOFT+’ condition), and no E-health support was available for parents (as was provided to parents in the ‘E-health4Uth Healthy Toddler’ condition).

### Measurements

Height and weight are measured at each YHC visit using standardized protocols [6], including height and weight at the visit at age 36 months; these data are collected from the YHC files. Parents are invited to complete questionnaires about overweight-related behaviors and determinants of these behaviors, and regarding health-related quality of life [24]. The questionnaires are provided to parents at inclusion (child circa 2 to 4 weeks old), and at child age 6, 13 and 36 months. YHC-professionals are invited to complete a brief questionnaire to gather information about their experiences regarding the care provided to the parents/children and regarding the specific intervention; this was done three times in the ‘BBOFT+’ and in the control group, and two times in the E-health4Uth group. Professionals in the ‘E-health4Uth Healthy Toddler’ intervention group are invited to complete a brief assessment form after the YHC-visit at age 18 and 24 months (i.e., process information and perceptions regarding the care).

The outcomes are measured at age 3 years; the measurements at inclusion, at child age 6 and 13 months can additionally be taken into account in the evaluation of the interventions. The primary outcome measures of the study are: (1) overweight inducing/reducing behaviors (i.e. activity and outdoor play, breakfast, drinking sweet beverages and watching television and devoting time to the computer and playing computer games; the ‘BBOFT+’ intervention also has a more healthy sleeping pattern as outcome; (2) Body Mass Index (BMI) and the prevalence of overweight and obesity. Children are categorized as overweight and obese based on internationally accepted gender and age-specific cut-off values for BMI [25].

Concerning the primary outcomes it is hypothesized that more favorable energy balance related behaviors (and a more healthy sleep pattern with regard to ‘BBOFT+’), a lower BMI and a lower proportion of children with overweight and obesity will be present in each intervention group, compared to the control group.

Secondary outcome measures are attitudes and other cognitive determinants of the parents regarding the health behaviors of their child, parenting styles and practices, and child health-related quality of life: (1) Attitudes, motivation and perceived control among parents regarding the four energy balance-related behaviors [24], (2) General parenting styles and parental practices and rules (these will be measured by Child rearing questionnaire for parents [26] and parenting practices and rules in relation to physical activity and food [27]), (3) Child health-related quality of life and wellbeing (Infant Toddler Quality of Life questionnaire - ITQOL) [24]). Concerning the secondary outcomes it is hypothesized that more favorable attitudes and other determinants of the parents regarding the overweight-related behaviors of their child will be present in each intervention group, compared to the control group. It is hypothesized that general parenting styles are less often restrictive and permissive, and more often authoritative in each intervention group compared to the control group, and that parenting practices and rules in relation to physical activity and food are more favorable in each intervention group, compared to the control group. It is hypothesized that child health-related quality of life and well-being are more favorable in each intervention group, compared to the control group.

We will explore whether one or more of the following variables have an effect on the relationship of interest, and if so, include them in the analyses as confounder: (1) Pregnancy duration and birth weight (from YHC files), (2) Parents’ BMI using self-report (self-report of adult height and weight has proven to be sufficiently accurate for epidemiological research [28]), (3) Parents’ health-related quality of life and wellbeing (SF-12), (4) Age, educational level and ethnic background of the parents.

In both intervention groups (i.e., ‘BBOFT+’ and the ‘E-health4Uth Healthy Toddler’) characteristics of the care process are measured via the questionnaires for parents at child age 6, 13 and 36 months [29]. YHC-professionals are invited to complete a brief questionnaire to gather information about their experiences regarding the care provided to the parents/children and regarding the specific intervention; this was done three times in the ‘BBOFT+’ and in the control group, and two times in the ‘E-health4Uth Healthy Toddler’ group. The questionnaires include: (1) Adherence of YHC professionals to the distinct elements of the intervention, (2) Appreciation/satisfaction of the interventions [29], and (3) Description of additional time investment. Professionals in the E-health4Uth Healthy Toddler intervention group are invited to complete a brief assessment form after the YHC-visit at age 18 and 24 months (i.e. care provided and perceptions regarding the care).

### Size of the study and power considerations

Fifty-one YHC teams of ten Youth Health Care providers participate in the study. They invited 3570 parents/children to participate in the study (two intervention groups and one control condition). Taking into account informed consent by 50% and dropout between baseline and follow-up of 30%, we expect complete data of 1250 parents/children at follow-up, equally divided over the two intervention groups and the control group. We assume equal standard deviations in the intervention groups and the control group, an alpha of 0.05 (2-tailed) and power of 0.80. We apply a correction factor to account for the cluster design, assuming an average cluster size of 25 toddlers (1250/51) and an intra-class correlation coefficient of 0.10. For this expected sample size and assumptions, we calculated minimal detectable differences between the intervention groups and the control group at follow-up.

Some illustrations: Regarding the mean number of the reported minutes of playing outside of the children per day, the study can detect a difference between an intervention group and the control group of 15 minutes/day (SD=52 min/day). Regarding the mean consumption of sweet drinks in glasses per day, a difference of 0.4 glasses/day (SD=1.5 glasses/day) can be detected. Regarding the mean number of minutes of TV viewing per day, a difference of 9 minutes/day can be detected (SD=32 minutes/day). BMI at age 36 months old: a difference of 0.38 BMI points between the mean BMI in an intervention group and the mean BMI in the control group can be detected by the study (SD=1.3).

### Data analyses

To assess the effect of both interventions, multilevel analyses will be applied to allow for dependency between the individual measurements within the YHC teams [30]. Multilevel linear and logistic regression analyses will be conducted for the continuous outcome variables with experimental group (i.e., intervention or control) as independent variable, and baseline values and relevant potential confounders, if any, as covariates. Multilevel logistic regression will be performed for dichotomous outcome variables. Additionally, by introducing an interaction term into the regression analyses, effect modification by gender, parental educational level, parent ethnic background, and parental overweight status will be explored. If any of these interaction terms reach statistical significance, stratified analyses will be performed.

## Discussion

In this paper the study protocol of a cluster randomized controlled trial regarding primary prevention of overweight and obesity in preschool children has been presented. The study will evaluate two overweight prevention interventions, of which the ‘BBOFT+’ intervention starts immediately after birth and the ‘E-health4Uth’ intervention starts at the age of circa 18 months. The interventions are offered by preventive Youth Health Care (YHC) centers in addition to the currently applied overweight prevention protocol for parents of preschool children in the Netherlands.

It is hypothesized that positive effects on various aspects of child lifestyles and BMI will be demonstrated among children in the two intervention groups in comparison to children in the control group. Several process characteristics including adherence and satisfaction by both parents and professionals will also be analyzed. This will provide insights relevant for future implementation, when the interventions prove to be successful.

Strengths of the study are the size of the study, and the randomizations of YHC teams (c-RCT design). Because the last measurement will be at child age 36 months, it will be possible to also investigate medium-term effects of the interventions (circa 1 year follow-up after the last E-health4Uth Healthy Toddler intervention). Another strength will be that the study is conducted in the setting of daily YHC practice, which will facilitate future implementation if the interventions are found to be effective. The participating YHC teams cover urban as well as rural areas, which will support generalization of the findings. A limitation of the study will be that the behaviors of the children and their parents are based on self-report by the parents.

In conclusion, this study will evaluate a protocol for the prevention of overweight and obesity in preschool children. The results of this study will provide insight into the effectiveness of two interventions that may be applied in child public health in the Netherlands.

## Competing interests

The authors declare that they have no competing interests.

## Authors’ contributions

HR, MH and MB-B, in collaboration with MB and SV, originated the idea for the study and its design and were responsible for acquiring the grant for the study. MS, EV, TR, SB and AG further developed the described study protocol. HR, MH, MB-B and MB supervise the study. All authors regularly participate in discussing the design and protocols used in the study. All authors have read and approved the final manuscript.

## Pre-publication history

The pre-publication history for this paper can be accessed here:

http://www.biomedcentral.com/1471-2458/13/974/prepub
